# Genetic and environmental variation in educational attainment: an individual-based analysis of 28 twin cohorts

**DOI:** 10.1038/s41598-020-69526-6

**Published:** 2020-07-29

**Authors:** Karri Silventoinen, Aline Jelenkovic, Reijo Sund, Antti Latvala, Chika Honda, Fujio Inui, Rie Tomizawa, Mikio Watanabe, Norio Sakai, Esther Rebato, Andreas Busjahn, Jessica Tyler, John L. Hopper, Juan R. Ordoñana, Juan F. Sánchez-Romera, Lucia Colodro-Conde, Lucas Calais-Ferreira, Vinicius C. Oliveira, Paulo H. Ferreira, Emanuela Medda, Lorenza Nisticò, Virgilia Toccaceli, Catherine A. Derom, Robert F. Vlietinck, Ruth J. F. Loos, Sisira H. Siribaddana, Matthew Hotopf, Athula Sumathipala, Fruhling Rijsdijk, Glen E. Duncan, Dedra Buchwald, Per Tynelius, Finn Rasmussen, Qihua Tan, Dongfeng Zhang, Zengchang Pang, Patrik K. E. Magnusson, Nancy L. Pedersen, Anna K. Dahl Aslan, Amie E. Hwang, Thomas M. Mack, Robert F. Krueger, Matt McGue, Shandell Pahlen, Ingunn Brandt, Thomas S. Nilsen, Jennifer R. Harris, Nicholas G. Martin, Sarah E. Medland, Grant W. Montgomery, Gonneke Willemsen, Meike Bartels, Catharina E. M. van Beijsterveldt, Carol E. Franz, William S. Kremen, Michael J. Lyons, Judy L. Silberg, Hermine H. Maes, Christian Kandler, Tracy L. Nelson, Keith E. Whitfield, Robin P. Corley, Brooke M. Huibregtse, Margaret Gatz, David A. Butler, Adam D. Tarnoki, David L. Tarnoki, Hang A. Park, Jooyeon Lee, Soo Ji Lee, Joohon Sung, Yoshie Yokoyama, Thorkild I. A. Sørensen, Dorret I. Boomsma, Jaakko Kaprio

**Affiliations:** 1 Department of Social Research, Faculty of Social Sciences, University of Helsinki, P.O. Box 18, 00014 Helsinki, Finland; 2 Center for Twin Research, Osaka University Graduate School of Medicine, Osaka, Japan; 3 Department of Physiology, Faculty of Medicine and Nursing, University of the Basque Country, Leioa, Spain; 4 Department of Public Health, University of Helsinki, Helsinki, Finland; 5 Institute of Clinical Medicine, University of Eastern Finland, Kuopio, Finland; 6 Faculty of Health Science, Kio University, Nara, Japan; 7 Department of Genetics, Physical Anthropology and Animal Physiology, University of the Basque Country UPV/EHU, Leioa, Spain; 8 HealthTwiSt GmbH, Berlin, Germany; 9 Twins Research Australia, Centre for Epidemiology and Biostatistics, The University of Melbourne, Melbourne, VIC Australia; 10 Department of Epidemiology, School of Public Health, Seoul National University, Seoul, Korea; 11 Department of Human Anatomy and Psychobiology, University of Murcia, Murcia, Spain; 12 IMIB-Arrixaca, Murcia, Spain; 13 Genetic Epidemiology Department, QIMR Berghofer Medical Research Institute, Brisbane, Australia; 14 Centre for Epidemiology and Biostatistics, Melbourne School of Population and Global Health, The University of Melbourne, Melbourne, Australia; 15 Pós-Graduação em Reabilitação e Desempenho Funcional, Universidade Federal dos Vales do Jequitinhonha e Mucuri, Diamantina, Brazil; 16 Musculoskeletal Health Research Group, Faculty of Health Sciences, The University of Sydney, Sydney, Australia; 17 Istituto Superiore di Sanità - Centre for Behavioural Sciences and Mental Health, Rome, Italy; 18 Centre of Human Genetics, University Hospitals Leuven, Leuven, Belgium; 19 Department of Obstetrics and Gynaecology, Ghent University Hospitals, Ghent, Belgium; 20 The Charles Bronfman Institute for Personalized Medicine, The Mindich Child Health and Development Institute, Icahn School of Medicine At Mount Sinai, New York, NY USA; 21 Institute of Research and Development, Battaramulla, Sri Lanka; 22 Faculty of Medicine and Allied Sciences, Rajarata University of Sri Lanka Saliyapura, Anuradhapura, Sri Lanka; 23 Institute of Psychiatry Psychology and Neuroscience, King’s College London, London, UK; 24 South London and Maudsley NHS Foundation Trust, London, UK; 25 Research Institute for Primary Care and Health Sciences, School for Primary Care Research (SPCR), Faculty of Health, Keele University, Staffordshire, UK; 26 Social Genetic and Developmental Psychiatry Research Centre, Institute of Psychiatry, Psychology and Neuroscience, King’s College London, London, UK; 27 Washington State Twin Registry, Washington State University - Health Sciences Spokane, Spokane, WA USA; 28 Department of Global Public Health, Karolinska Institutet, Stockholm, Sweden; 29 Epidemiology, Biostatistics and Biodemography, Department of Public Health, University of Southern Denmark, Odense, Denmark; 30 Department of Public Health, Qingdao University Medical College, Qingdao, China; 31 Department of Noncommunicable Diseases Prevention, Qingdao Centers for Disease Control and Prevention, Qingdao, China; 32 Department of Medical Epidemiology and Biostatistics, Karolinska Institutet, Stockholm, Sweden; 33 Institute of Gerontology and Aging Research Network – Jönköping (ARN-J), School of Health and Welfare, Jönköping University, Jönköping, Sweden; 34 Department of Preventive Medicine, Keck School of Medicine of USC, University of Southern California, Los Angeles, CA USA; 35 USC Norris Comprehensive Cancer Center, Los Angeles, CA USA; 36 Department of Psychology, University of Minnesota, Minneapolis, MN USA; 37 Department of Psychology, University of California, Riverside, Riverside, CA 92521 USA; 38 Division of Health Data and Digitalization, Norwegian Institute of Public Health, Oslo, Norway; 39 Institute for Molecular Bioscience, The University of Queensland, Brisbane, Australia; 40 Department of Biological Psychology, Netherlands Twin Register, Vrije Universiteit, Amsterdam, Amsterdam, The Netherlands; 41 Department of Psychiatry, University of California, San Diego, CA USA; 42 VA San Diego Center of Excellence for Stress and Mental Health, La Jolla, CA USA; 43 Department of Psychological and Brain Sciences, Boston University, Boston, MA USA; 44 Department of Human and Molecular Genetics, Virginia Institute for Psychiatric and Behavioral Genetics, Virginia Commonwealth University, Richmond, VA USA; 45 Department of Human and Molecular Genetics, Psychiatry and Massey Cancer Center, Virginia Commonwealth University, Richmond, VA USA; 46 Department of Psychology, University of Bremen, Bremen, Germany; 47 Department of Health and Exercise Sciences and Colorado School of Public Health, Colorado State University, Fort Collins, USA; 48 Psychology Department, Wayne State University, Detroit, MI USA; 49 Institute for Behavioral Genetics, University of Colorado, Boulder, CO USA; 50 Institute of Behavioral Science, University of Colorado, Boulder, CO USA; 51 Center for Economic and Social Research, University of Southern California, Los Angeles, CA USA; 52 Health and Medicine Division, The National Academies of Sciences, Engineering, and Medicine, Washington, DC USA; 53 Medical Imaging Centre, Semmelweis University, Budapest, Hungary; 54 Hungarian Twin Registry, Budapest, Hungary; 55 Department of Emergency Medicine, Hallym University Dongtan Sacred Heart Hospital, Hwaseong, Korea; 56 Institute of Health and Environment, Seoul National University, Seoul, South Korea; 57 Department of Public Health Nursing, Osaka City University, Osaka, Japan; 58 Novo Nordisk Foundation Centre for Basic Metabolic Research (Section of Metabolic Genetics), Faculty of Health and Medical Sciences, University of Copenhagen, Copenhagen, Denmark; 59 Department of Public Health (Section of Epidemiology), Faculty of Health and Medical Sciences, University of Copenhagen, Copenhagen, Denmark; 60 Institute for Molecular Medicine Finland FIMM, University of Helsinki, Helsinki, Finland

**Keywords:** Genetics, Psychology

## Abstract

We investigated the heritability of educational attainment and how it differed between birth cohorts and cultural–geographic regions. A classical twin design was applied to pooled data from 28 cohorts representing 16 countries and including 193,518 twins with information on educational attainment at 25 years of age or older. Genetic factors explained the major part of individual differences in educational attainment (heritability: a^2^ = 0.43; 0.41–0.44), but also environmental variation shared by co-twins was substantial (c^2^ = 0.31; 0.30–0.33). The proportions of educational variation explained by genetic and shared environmental factors did not differ between Europe, North America and Australia, and East Asia. When restricted to twins 30 years or older to confirm finalized education, the heritability was higher in the older cohorts born in 1900–1949 (a^2^ = 0.44; 0.41–0.46) than in the later cohorts born in 1950–1989 (a^2^ = 0.38; 0.36–0.40), with a corresponding lower influence of common environmental factors (c^2^ = 0.31; 0.29–0.33 and c^2^ = 0.34; 0.32–0.36, respectively). In conclusion, both genetic and environmental factors shared by co-twins have an important influence on individual differences in educational attainment. The effect of genetic factors on educational attainment has decreased from the cohorts born before to those born after the 1950s.

## Introduction

Education is one of the most important intellectual, social and material resources in modern societies. It typically precedes occupation, strongly affects occupation-based social position and lifetime income^1^, and is associated with health and mortality^2^. The education system is also the main institution reproducing social stratification^3^. Accordingly, the understanding of how genetic and environmental factors underlie the individual differences in educational attainment has not only scientific but also important societal implications. A meta-analysis of twin studies conducted in 10 countries found that genetic and environmental factors shared by co-twins explain about equal shares (around 40%) of the inter-individual differences in educational level^4^. The proportion of contribution by shared environment was substantially higher than found in a meta-analysis of performance in school subjects^5^. The shared environmental variation in intelligence is still substantial in childhood, but diminishes until adulthood^6^. Thus, in addition to cognitive skills, childhood family and other environmental factors seem to affect educational level. A previous study demonstrated the importance of social inheritance by showing that good parental socio-economic position is associated with academic success even after adjusting for cognitive ability^7^.

The role of genetic and environmental factors in educational differences can also vary between societies and changes over time because of differences between educational systems and societies in general^4,5^. For example, in more meritocratic and open societies, the role of individual differences derived from genetic factors may be more pronounced, whereas in socially more closed societies with less social mobility, childhood social factors may be more important. Information on how the macro-environment modifies the role of genetic and environmental factors behind inter-individual variation in education is important for the understanding of how socially and politically established barriers in the society modify the realization of genetic potential for educational outcomes. An early Norwegian twin study found that the role of genetic factors on education increased after World War II when the educational system was reformed^8^, and a study in Spain showed similar results on the increasing effect of genetic factors on education after an educational reform promoting equality in educational opportunities^9^. This is further supported by a re-analysis of the published heritability estimates of educational attainment^4^ finding that high intergenerational mobility in a society was associated with the higher proportion of genetic and the lower proportion of shared environmental factors of the total variation of educational attainment^10^. These twin study results correspond well with the findings that the effect of parental socioeconomic position on offspring education has diminished over the recent decades in Europe^11^. The previous literature thus suggests that a more open society, in which educational reforms make the education system more open and meritocratic, will strengthen the influence of genetic factors and decrease the influence of family background on educational attainment. An Estonian study supported this conclusion by showing that previously identified genetic polymorphisms associated with educational attainment explained a larger proportion of variation in educational attainment in the cohorts who received their education after rather than before the independence of Estonia from the Soviet Union^12^.

In this study, we analyzed the contribution of genetic and environmental factors to educational attainment in a large multinational twin database. Based on previous twin studies^8,9^, we expect that the role of genetic factors in educational variation has increased over the recent decades, especially after World War II when the educational system was expanded in many countries. We further expect that the increasing general level of education may increase the proportion of genetic variation since the decision for higher education is done later in adolescence when the children have become more independent from their parents^13^. Societies also differ whether education is free for students or whether tuitions are needed which may affect how parental financial resources affect possibilities for higher education. Thus, we expect to see macro-level differences between countries, which may reflect access to higher education. We classified the countries in accordance with the typology of welfare regimes presented by Esping-Andersen^14^; European countries follow the social democratic, conservative or mixed societal models whereas the USA and Australia follow the liberal model. The East Asian countries were difficult to classify by this typology, but they share many cultural similarities. Finally, we will analyze gender differences in the proportions of genetic and environmental variation in educational attainment, and whether they have changed over the twentieth century when opportunities for higher education have equalized for men and women.

## Results

Table 1 presents the descriptive statistics in the whole data as well as stratified by cultural–geographic region (the corresponding statistics by twin cohort are available in Supplementary Table 1). The educational level increased over the birth cohorts in men and women: when the most recent birth cohorts (1980–1989), where education may not yet have been finished, were removed, the increase was 0.55 (95% confidence interval (CI) 0.53–0.57) educational years per decade in men and 0.92 (95% CI 0.90–0.94) educational years per decade in women. Mean education was virtually the same in men and women (12.5 years) in the whole dataset, but the educational level increased more rapidly in women than in men (0.37 educational years more per decade in women than in men; 95% CI 0.35–0.40). In all birth cohorts, the educational level was higher in North America and Australia than in Europe or East Asia.

**Table 1:** Number of twins and means and standard deviations (SD) of education by cultural–geographic region and gender.

	All^a^			Europe^b^			North America and Australia^c^			East Asia^d^		
	N	Mean	SD	N	Mean	SD	N	Mean	SD	N	Mean	SD
**Men**												
1900–1909	569	8.5	4.67	393	6.0	2.75	176	13.8	3.40	na	–	–
1910–1919	3823	11.1	4.27	1692	8.3	3.72	2131	13.3	3.25	na	–	–
1920–1929	14,651	12.2	4.07	4801	9.0	4.02	9824	13.7	3.08	na	–	–
1930–1939	10,556	10.8	4.45	7815	9.6	4.25	2693	14.3	2.78	na	–	–
1940–1949	20,382	11.8	4.38	13,824	10.4	4.37	6455	14.8	2.56	na	–	–
1950–1959	20,590	13.0	3.69	11,168	11.7	4.04	9110	14.6	2.40	182	10.9	4.32
1960–1969	7807	13.9	2.92	1420	12.2	3.69	5904	14.6	2.30	278	12.3	3.74
1970–1979	8571	14.2	2.79	4528	14.1	2.74	3253	14.8	2.25	483	13.3	4.06
1980–1989	2961	14.2	2.69	783	13.5	2.96	1805	14.9	2.09	55	14.5	4.16
**Women**												
1900–1909	1025	8.6	4.49	650	6.0	2.61	375	13.1	3.38	na	–	–
1910–1919	3907	10.0	4.10	2342	7.8	3.24	1565	13.2	2.99	na	–	–
1920–1929	8593	10.3	4.03	5504	8.6	3.65	3080	13.4	2.67	na	–	–
1930–1939	11,626	10.7	4.09	8266	9.6	4.07	3326	13.5	2.53	na	–	–
1940–1949	23,391	11.8	4.03	15,003	10.6	4.17	8223	14.1	2.42	na	–	–
1950–1959	24,852	13.1	3.54	12,663	12.1	4.01	11,776	14.4	2.33	228	10.9	4.04
1960–1969	12,393	14.0	2.84	2514	12.6	3.60	9183	14.6	2.25	424	12.3	3.64
1970–1979	12,683	14.7	2.70	5386	14.5	2.76	6160	15.2	2.21	744	14.1	3.76
1980–1989	4877	14.7	2.59	1354	14.4	2.82	3018	15.2	2.09	115	13.7	3.44

^a^Twins cohorts in footnotes 2–4 and additionally Sri Lanka Twin Registry and Brazilian Twin Registry.

^b^Berlin Twin Register Health, Bielefeld Longitudinal Study of Adult Twins, Netherlands Twin Cohort, Finnish Older Cohort, FinnTwin12, FinnTwin16, East Flanders Prospective Twin Survey, Hungarian Twin Registry, Italian Twin Registry, Murcia Twin Registry, Norwegian Twin Registry, Swedish Young Male Twins Study, Swedish Twin Cohorts.

^c^Twins Research Australia (formerly Australia Twin Registry), California Twin Program, Carolina African American Twin Study of Aging, Colorado Twin Registry, Mid Atlantic Twin Registry, Minnesota Twin Registry, Queensland Twin Register, Washington State Twin Registry, Vietnam Era Twin Registry, NAS-NRC twin cohort.

^d^Korean Twin-Family Register, Osaka University Aged Twin Registry, Qingdao Twin Registry.

Table 2 presents the proportions of educational variation explained by genetic and environmental factors. In the whole data, the largest proportion of individual differences in education was explained by genetic factors (heritability: a^2^ = 0.43; 95% CI 0.41–0.44), but environmental factors shared by co-twins were also substantial (c^2^ = 0.31; 95% CI 0.30–0.33). When stratified by gender, we found that the proportion of variation in educational attainment attributable to genetic factors was larger in men than in women (a^2^ = 0.47; 95% CI 0.45–0.50 vs. a^2^ = 0.38; 95% CI 0.36–0.40) – and correspondingly the proportion explained by shared environmental factors smaller (c^2^ = 0.26; 95% CI 0.24–0.29 vs. c^2^ = 0.36; 95% CI 0.34–0.38). The highest heritability was found in the latest birth cohort born in 1980–1989 (a^2^ = 0.60; 95% CI 0.50–0.71). This result remained when we restricted the analyses only to those of 30 years of age or older to confirm that that this was not affected by unfinished education in this birth cohort (Supplementary Table 2).

**Table 2:** The proportions of educational variation explained by additive genetic, shared environmental and unique environmental variances with 95% confidence intervals by birth cohort and gender.

	Additive genetic factors			Shared environment			Unique environment		
	a^2^	95% CI		c^2^	95% CI		e^2^	95% CI	
		LL	UL		LL	LL		LL	UL
**Men and women**									
All	0.43	0.41	0.44	0.31	0.30	0.33	0.26	0.26	0.26
1900–1909	0.12	0.00	0.25	0.62	0.51	0.72	0.26	0.21	0.31
1910–1919	0.37	0.30	0.44	0.42	0.36	0.48	0.21	0.19	0.23
1920–1929	0.43	0.39	0.48	0.33	0.29	0.37	0.24	0.22	0.25
1930–1939	0.41	0.36	0.46	0.32	0.27	0.36	0.27	0.26	0.29
1940–1949	0.47	0.43	0.50	0.27	0.23	0.30	0.27	0.26	0.28
1950–1959	0.40	0.37	0.43	0.35	0.32	0.37	0.26	0.25	0.26
1960–1969	0.27	0.22	0.33	0.39	0.33	0.43	0.34	0.33	0.36
1970–1979	0.34	0.29	0.40	0.35	0.29	0.40	0.31	0.29	0.32
1980–1989	0.60	0.50	0.71	0.13	0.03	0.23	0.26	0.25	0.28
**Men**									
All	0.47	0.45	0.50	0.26	0.24	0.29	0.27	0.26	0.27
1900–1909	0.33	0.15	0.54	0.49	0.28	0.65	0.18	0.13	0.25
1910–1919	0.48	0.38	0.59	0.32	0.21	0.41	0.20	0.18	0.23
1920–1929	0.45	0.40	0.51	0.31	0.25	0.35	0.24	0.23	0.26
1930–1939	0.41	0.34	0.48	0.31	0.25	0.38	0.28	0.26	0.30
1940–1949	0.54	0.49	0.60	0.19	0.14	0.24	0.27	0.25	0.28
1950–1959	0.41	0.36	0.46	0.31	0.26	0.35	0.28	0.27	0.30
1960–1969	0.32	0.23	0.42	0.36	0.27	0.44	0.32	0.30	0.35
1970–1979	0.36	0.27	0.46	0.32	0.23	0.40	0.32	0.30	0.35
1980–1989	0.63	0.46	0.75	0.09	0.00	0.25	0.28	0.25	0.32
**Women**									
All	0.38	0.36	0.40	0.36	0.34	0.38	0.26	0.25	0.26
1900–1909	0.05	0.00	0.17	0.69	0.58	0.76	0.26	0.21	0.31
1910–1919	0.24	0.16	0.33	0.55	0.46	0.62	0.21	0.19	0.24
1920–1929	0.39	0.32	0.46	0.39	0.33	0.45	0.22	0.20	0.24
1930–1939	0.41	0.34	0.48	0.32	0.26	0.38	0.27	0.25	0.29
1940–1949	0.39	0.34	0.44	0.35	0.30	0.39	0.27	0.25	0.28
1950–1959	0.38	0.34	0.42	0.38	0.35	0.42	0.24	0.23	0.25
1960–1969	0.25	0.18	0.32	0.40	0.34	0.46	0.35	0.33	0.37
1970–1979	0.33	0.27	0.40	0.37	0.30	0.43	0.30	0.28	0.32
1980–1989	0.58	0.46	0.72	0.16	0.03	0.28	0.26	0.24	0.28

We then conducted birth-cohort-specific analyses by the cultural–geographic region (Table 3). The three cultural–geographic regions did not show systematic differences in the proportions of genetic and environmental factors. When pooling all birth cohorts together, genetic factors explained virtually the identical proportions of individual differences in educational attainment in Europe (a^2^ = 0.40; 95% CI 0.37–0.42) as in North America and Australia (a^2^ = 0.39; 95% 0.36–0.41). In East Asia, the heritability point estimate was lower but did not differ statistically significantly from the other cultural–geographic regions either (a^2^ = 0.32; 95% 0.19–0.48); because of the smaller sample size, the confidence interval was wide. When we stratified the results by gender, there were no differences between the cultural–geographic regions (Supplementary Table 3 for men and 4 for women).

**Table 3:** The proportions of educational variation explained by additive genetic, shared environmental and unique environmental variances with 95% confidence intervals by birth cohort and cultural–geographic region in men and women.

	Additive genetic factors			Shared environment			Unique environment		
	a^2^	95% CI		c^2^	95% CI		e^2^	95% CI	
		LL	UL		LL	LL		LL	UL
**Europe**									
All	0.40	0.37	0.42	0.35	0.33	0.37	0.26	0.25	0.26
1900–1909	0.21	0.05	0.36	0.58	0.44	0.70	0.22	0.17	0.28
1910–1919	0.34	0.24	0.44	0.42	0.33	0.50	0.25	0.21	0.28
1920–1929	0.30	0.23	0.37	0.44	0.38	0.50	0.26	0.24	0.28
1930–1939	0.34	0.28	0.40	0.36	0.31	0.41	0.29	0.27	0.31
1940–1949	0.43	0.38	0.47	0.30	0.26	0.34	0.27	0.26	0.29
1950–1959	0.40	0.36	0.44	0.37	0.33	0.40	0.24	0.22	0.25
1960–1969	0.31	0.18	0.45	0.36	0.23	0.48	0.33	0.30	0.36
1970–1979	0.48	0.40	0.57	0.23	0.14	0.31	0.29	0.27	0.31
1980–1989	0.69	0.48	0.75	0.02	0.00	0.22	0.29	0.25	0.33
**North America and Australia**									
All	0.39	0.36	0.41	0.32	0.30	0.29	0.29	0.28	0.30
1900–1909	0.17	0.00	0.41	0.55	0.33	0.72	0.28	0.21	0.37
1910–1919	0.47	0.38	0.57	0.34	0.24	0.43	0.19	0.17	0.21
1920–1929	0.50	0.44	0.55	0.26	0.21	0.32	0.24	0.23	0.25
1930–1939	0.43	0.34	0.53	0.29	0.19	0.38	0.28	0.26	0.30
1940–1949	0.42	0.35	0.48	0.29	0.23	0.35	0.30	0.28	0.31
1950–1959	0.33	0.28	0.38	0.34	0.29	0.39	0.33	0.31	0.34
1960–1969	0.29	0.23	0.36	0.35	0.29	0.41	0.36	0.34	0.38
1970–1979	0.21	0.14	0.30	0.43	0.36	0.50	0.35	0.33	0.38
1980–1989	0.59	0.45	0.72	0.12	0.00	0.25	0.29	0.27	0.32
**East Asia**									
All	0.32	0.19	0.48	0.43	0.28	0.56	0.24	0.22	0.27
1960–1969	0.21	0.03	0.46	0.55	0.30	0.72	0.24	0.19	0.29
1970–1979	0.15	0.00	0.35	0.55	0.35	0.70	0.30	0.26	0.35
1980–1989	0.74	0.37	0.83	0.00	0.00	0.33	0.26	0.17	0.40

Table 4 presents the analyses stratifying cohorts born in 1900–1949 and 1950–1989 in participants at 30 years of age or older from European, North American and Australian twin cohorts together. The role of genetic factors decreased (a^2^ = 0.44; 95% CI 0.41–0.46 vs. a^2^ = 0.38; 95% CI 0.36–0.40) and the role of shared environment increased over the birth cohorts (c^2^ = 0.31; 95% CI 0.29–0.33 vs. c^2^ = 0.34; 95% CI 0.32–0.36). When we stratified these analyses into Europe and North America and Australia, we saw a similar decrease in the proportion of additive genetic factors and increase in the proportion of shared environmental factors in North America and Australia. In the gender-stratified analyses, women showed only minor decrease in the role of additive genetic factors. Thus, the difference between men and women in the proportion of variation explained by additive genetic factors was greater in the cohorts born in 1900–1949 than in 1950–1989 (a^2^ = 0.49; 95% CI 0.46–0.52 vs. a^2^ = 0.41; 95% CI 0.37–0.44 in men and a^2^ = 0.38; 95% CI 0.35–0.41 vs. a^2^ = 0.36; 95% CI 0.32–0.39 in women).

**Table 4:** The proportions of educational variation explained by additive genetic, shared environmental and unique environmental variances with 95% confidence intervals in European, North American and Australian men and women born in 1900–1949 and 1950–1989.

	Additive genetic factors			Shared environment			Unique environment		
	a^2^	95% CI		c^2^	95% CI		e^2^	95% CI	
		LL	UL		LL	LL		LL	UL
**All men and women**									
1900–1949	0.44	0.41	0.46	0.31	0.29	0.33	0.25	0.25	0.26
1950–1989	0.38	0.36	0.40	0.34	0.32	0.36	0.28	0.27	0.29
**European men and women**									
1900–1949	0.37	0.34	0.40	0.36	0.33	0.39	0.27	0.26	0.28
1950–1989	0.41	0.38	0.45	0.33	0.30	0.36	0.26	0.25	0.27
**North American and Australian men and women**									
1900–1949	0.45	0.41	0.49	0.29	0.26	0.33	0.26	0.25	0.26
1950–1989	0.32	0.29	0.36	0.35	0.31	0.38	0.33	0.32	0.34
**All men**									
1900–1949	0.49	0.46	0.52	0.26	0.23	0.29	0.25	0.24	0.26
1950–1989	0.41	0.37	0.44	0.30	0.27	0.34	0.29	0.28	0.30
**All women**									
1900–1949	0.38	0.35	0.41	0.37	0.34	0.40	0.25	0.24	0.26
1950–1989	0.36	0.32	0.39	0.37	0.34	0.40	0.27	0.26	0.28
**European men**									
1900–1949	0.42	0.37	0.46	0.30	0.26	0.34	0.29	0.27	0.30
1950–1989	0.36	0.29	0.42	0.35	0.29	0.40	0.29	0.28	0.32
**European women**									
1900–1949	0.32	0.28	0.36	0.42	0.38	0.45	0.26	0.25	0.28
1950–1989	0.40	0.35	0.45	0.37	0.32	0.41	0.23	0.22	0.25
**North American and Australian men**									
1900–1949	0.56	0.52	0.60	0.20	0.16	0.23	0.25	0.23	0.26
1950–1989	0.34	0.28	0.40	0.34	0.28	0.39	0.32	0.31	0.34
**North American and Australian women**									
1900–1949	0.35	0.29	0.41	0.38	0.32	0.43	0.27	0.26	0.29
1950–1989	0.31	0.25	0.36	0.34	0.29	0.39	0.36	0.34	0.37

Restricted to participants 30 years of age or older.

Finally, we applied the genetic models separately to each twin cohort to explore whether there was a consistent, regular pattern within the countries not captured by our classification of the three cultural–geographic regions (Supplementary Table 5). These analyses showed no systematic differences in the estimates of proportions of genetic and environmental variation between the cohorts. Confidence intervals, however, were wide in many cohorts making it difficult to draw firm conclusions. When stratifying the analyses by gender (Supplementary Tables 6 and 7), these cohort specific analyses confirmed the higher heritability estimates in men than in women: only in six out of 24 cohorts including both genders, the point estimates of heritability were higher in women than in men.

## Discussion

In this dataset, pooling twin cohorts from 16 countries, we found that genetic factors explained 43% of the individual differences in educational attainment. The role of genetic factors affecting differences in educational attainment is not surprising. Intelligence, having an important effect on educational attainment, shows a substantial influence of genetic factors^6^, but there are also other genetic factors, which may affect educational attainment, for example, through personality aspects such as conscientiousness^15^. Genetic factors also contribute to the choice of subject at school, which can affect further opportunities for higher education^16^. The latest meta-analysis of genome-wide association (GWA) studies, including 1.1 million participants, identified 1271 genome-wide significant loci associated with education, but even together, they explained only 3.86% of the variation of education^17^. When using all common genetic variants (known as SNP-heritability), 12% of educational variation could be explained^18^. This lower proportion of genetic variation estimated by measured genetic polymorphisms than in our estimates based on twin design reflects the multifactorial and polygenic background of educational attainment; the current GWA technology does not capture all existing genetic variation.

In addition to genetic factors, our results show the importance of shared environmental factors explaining 31% of educational variation. This variation can include the effect of home environment but also the influence of school, common peers and other environmental factors shared by co-twins. This result is interesting since many previous studies have reported that shared environmental influences on intelligence^6^ or personality factors are small or not existing in adulthood^19^. Further, studies have not shown shared environmental effects for other socio-economic traits, such as income^20^. It is, however, noteworthy that disentangling genetic and shared environmental effects needs considerable statistical power^21^, which may have led to ignoring shared environmental effects in some smaller studies. A large Swedish twin study of young adult men found that shared environmental factors explained 20% of variation of intelligence^22^. Furthermore, differences in intelligence in childhood, influenced by greater shared environmental effect, may be more important for education than differences in adult intelligence. However, we found the substantial shared environmental variance components in nearly all twin cohorts, which were larger than generally found for intelligence, even in childhood^6^. These estimates were also higher than found in previous studies for academic performance at school^5^. Previous results have shown that parental socio-economic position affects offspring education, even after adjusting the results for cognitive ability^7^. A recent study demonstrated that the alleles of the educational polygenic score of parents that were not transmitted to their offspring were associated with school performance at age 17^23^ as well as the educational years in adulthood in offspring^24^. This genetic nurture effect was replicated in a Dutch study, where in adults, both transmitted and non-transmitted alleles included in educational polygenic scores were associated with offspring education^25^. In a UK study, polygenic education scores predicted school performance in adolescence better between individuals from different families than within dizygotic (DZ) co-twins, suggesting that a part of the genetic effect on education was because of family environment created by parental genotype^26^. Together, these results support the conclusion that even when family environment has a limited effect on many psychological and social outcomes^27^, it may be important for education. However, more research is needed to reveal the mechanisms between family background and education.

We found that genetic factors explained a larger proportion, and shared environmental factors a smaller proportion, of educational variation in the earlier born cohorts in 1900–1949 than in the later born cohorts in 1950–1989. These results, thus, do not support our initial hypothesis that the role of genetic factors declined over the birth cohorts because of higher educational level, especially in women, reflecting the expansion of higher education after World War II, which may have led to higher meritocracy. There have been somewhat contrasting results on the intergenerational transmission of education, one study suggesting diminishing effect of parental social-position in eight European countries^11^ and another study showing no change in the correlation between parental and offspring education in 42 countries^28^. The previous meta-analysis on the heritability of education, including several twin cohorts included also in our study, also found decreasing shared environmental variation in the cohorts born after 1950, but this effect was not statistically significant^4^. We confirmed these results by limiting these analyses to those of 30 years of age or older having thus virtually finished their education. Thus, unfinished education does not affect the results, which may happen, for example, if those with higher intelligence have finished their education in a shorter period.

Our region-specific results are also against of our prior hypothesis. We expected that shared environment would be especially large in countries following the liberal model of typology by Esping-Andersen^14^, i.e. North America and Australia in this study, leading to lower heritability, because in these countries parental economic resources may be more important for higher education than in the countries where higher education is free and organized by the government. However, this was not the case: the heritability estimates were at the same level as in the European countries including in this study countries following the social-democratic or conservative models. We analyzed this in more detail in individual cohorts to assess whether there would be a systematic pattern not captured by our classification of countries. However, we did not find any evidence on systematic differences in the heritability estimates between the countries. Our results suggest that shared environment has an important role in educational differences regardless of the model of society. A study showing a higher genetic effect in Estonia after re-independence from the Soviet Union indicates that the societal structure can play a role in the genetic variation of education^12^. However, it may be apparently limited to specific societal systems, such as former Soviet Union, or specific times in a societal development.

Our most consistent result was the larger proportion of genetic variation and consequently the smaller proportion of shared environmental variation of education in men than in women. This supports a previous meta-analysis, based mainly on published results^4^, but is extended with a greater number of countries, thus showing the universality of the result. Importantly, there is no evidence on a gender difference in the role of genetic and shared environmental factors in intelligence^29^. This suggests that family background and other environmental factors shared by co-twins are more important for women than for men in choosing to continue education. Interestingly, the differences in the heritability estimates between men and women were larger in the cohorts born in 1900–1949 than in the later cohorts born in 1950–1989 when the role of shared environment increased in men. Thus, in the later birth cohorts, the role of genetic and environmental factors converged in men and women. Parallel results have been found in molecular genetic research: A US study found that the polygenic risk score of education predicted educational years more strongly in men than in women and this difference has diminished from the cohorts born in the 1930s to 1950s^30^. This may be associated to more equal opportunities for women to get a higher education in the later birth cohorts, which also this study showed by an increasing education in women.

One factor which may have inflated the estimates of shared environmental variation is assortative mating. There is a well-known spousal correlation in education, which may itself change over time^31^. If this induces a genetic correlation between spouses, the genetic correlation between DZ twins becomes higher than 0.5 as assumed by the twin model. In a sub-cohort, we had information on maternal and paternal education (23,705 families). The spousal correlation in the parents of twins was 0.57 after adjusting for the twin cohort and the birth year of twin children used as a proxy of the missing information on the birth years of parents. If applied to the estimates of genetic and environmental variation using the formula presented by Martin^32^, this spousal correlation was too high even if all common environmental variation is caused by assortative mating, i.e., it should have produced even higher shared environmental variance component than we found. However, in addition to phenotypic assortment, social homogamy, i.e. similar social environment of spouses, can affect a spousal correlation, which does not generate a correlation between genotypes of spouses^33^. A US study found that the correlation of polygenic risk score of education was 0.13 between spouses, which was much lower than the spousal educational correlation^34^. Correcting shared environmental variation by this genetic correlation reduced them somewhat, but they remained substantial (c^2^ = 0.19 in men and c^2^ = 0.30 in women). However, the polygenic risk score explains only a fraction of educational variation^17^, and so we should make an assumption that the spouses share the same amount of unknown genetic variants affecting education than they share the known genetic variants. In a UK study using all genetic loci affecting education, the genetic correlation between spouses was even higher (r_A_ = 0.65) than the educational trait correlation^35^. When applied to our estimates, this genetic correlation should have produced even higher shared environmental variation than found in this study. The higher genetic than trait correlation in education would also expect a different mechanism behind assortative mating, i.e. selecting a spouse because of genetic liability rather than the trait itself, than previously assumed. Thus, it is too early to argue how much assortative mating has inflated shared environmental variation in this study because we do not know the mechanism behind assortment well enough to be able to estimate the genetic spousal correlation. However, we found that there were no differences in spousal correlations between cohorts born 1900–1949 and 1950–1989 (r = 0.59 vs. r = 0.54, respectively) or, as expected, between the parents of males and females (r = 0.58 vs. r = 0.55, respectively) which could explain the differences in the genetic architecture between birth cohorts or between men and women. In contrast, the spousal correlations were thus even lower in the latter birth cohorts and in women, which thus cannot explain the larger shared environmental variation in these groups than in the earlier birth cohorts and men.

Our data have important strengths but also weaknesses. As compared to the previous meta-analysis of twin studies of education based mainly on published results^4^, our study based solely on individual level data offered several important strengths. With access to individual data, we could conduct more flexible analyses than could be done when based on published estimates. Further, our data are free of publication bias, which may lead to tendency to publish results in line of previously published heritability estimates, and we also have more data from a larger number of countries, including Asian countries, than in this previous meta-analysis^4^. The progress of GWA studies have allowed to estimate the role of genetic factors based on individual level data with information on common genetic variants^17^; GWA studies, however, lack information on rarer variants and the more complex genetic variation that is captured by whole genome sequencing. A strength of the classical twin design is that it allows separating the effects of all genetic and of environmental factors shared by co-twins, such as family background. Thus, twin studies allow for a more comprehensive understanding of how genetic and social inheritance affect the transmission of education across generations. A weakness of our data is that we have information only on accomplished education. It would be important to collect also information on school performance, intelligence, motivation and other factors affecting education. This would help to understand mechanisms behind genetic and environmental components of educational variation.

In conclusion, our international twin data demonstrated that genetic factors are important for educational attainment regardless of the society. We also found environmental variation shared by co-twins behind inter-individual differences in educational attainment, but its estimate depends how we assume assortative mating to affect the genetic correlation between spouses. The role of shared environmental variation was higher in women than in men, especially in the cohorts born before World War II. This suggests that the influence of environmental factors on education differs between men and women and this gender difference itself may change over time.

## Data and methods

### Sample

The data for this study were derived from the international COllaborative project of Development of Anthropometrical measures in Twins (CODATwins) database. The CODATwins project aimed to pool data from all twin cohorts in the world having information on height and weight^36^. However, additional information was also collected on own education^37^. Together, 28 twin cohorts representing 16 countries provided data on education to the CODATwins database and were included in the present study. The footnote of Table 1 shows the names of these cohorts. Among these cohorts, 13 come from Europe, 8 from the USA, two from Australia and single cohorts from South Korea, Japan, China, Brazil and Sri Lanka (Supplementary Table 1). We classified the countries in accordance with the typology of welfare regimes presented by Esping-Andersen^14^: European countries follow the social democratic (Finland, Norway and Sweden), conservative (German, Hungarian, Italy and Spain) or mixed (Netherlands and Belgium) societal models whereas the USA and Australia follow the liberal model. The East Asian countries (China, Japan and South Korea) were difficult to classify by this typology, but they share many cultural similarities. Sri Lanka and Brazil were not included in these region-specific analyses, because they are geographically and culturally distinct from the other regions.

All participants were volunteers and gave informed consent when participating in their original study. Only a limited set of observational variables and anonymized data were delivered to the data management center at University of Helsinki. The pooled analysis was approved by the ethical committee of Department of Public Health, University of Helsinki, and the methods were carried out in accordance with the approved guidelines.

We transformed the different educational classifications used in the original surveys from each cohort into educational years by using the mean levels of educational years in each category. The original classifications and the corresponding numbers of educational years have been described elsewhere^37^. To ensure that the educational attainment variable captured the participants’ final highest level of education, we removed those under 25 years of age from the sample. We used this age limit because a higher age limit would have led to a substantial loss of data in the latest birth cohorts. However, when making comparisons between birth cohorts, we used a stricter age limit, i.e. 30 years, to confirm that unfinished education would not affect the results. Further, we replicated the heritability analyses by birth cohort using this stricter age limit to analyze whether it would change the results systematically. Together, we had educational data from 193,518 twins (54% women) including 81,894 complete twin pairs; 40% of the complete twin pairs were monozygotic (MZ), 39% same-sex dizygotic (SSDZ) and 21% opposite-sex dizygotic (OSDZ). From these participants, 39,235 were under 30 years of age, and we thus removed them when using this stricter age limit. The birth years of the twins ranged from 1889 to 1989. However, we had data for only 70 men and 137 women born earlier than 1900, whom we removed from the analyses because the number was too small for birth cohort specific analyses. The largest number of twins were from Europe (N = 100,272) and North America and Australia (N = 88,106), while the number of twins was much smaller for East Asia (N = 2728). In the region-specific analyses, East Asians born before 1950 (113 men and 106 women) were removed because the number was too small for the analyses stratified by birth cohort and region.

### Statistical analyses

The data were analyzed using quantitative genetic twin modeling based on structural equation models^38^. The twin modeling uses the different genetic relatedness of MZ and DZ twins: DZ twins share, on average, 50% of genetic variation, whereas MZ twins are virtually identical at the DNA sequence level. This principle allows decomposing the educational variation into variance attributable to additive genetic factors (A: correlated by 1.0 for MZ and 0.5 for DZ pairs), environmental factors shared by co-twins (C: by definition correlated by 1.0 for both MZ and DZ pairs), and environmental factors unique to each twin individual (E: by definition uncorrelated for MZ and DZ pairs). The unique environmental component also includes measurement error. The models were estimated using the OpenMx package (version 2.0.1) of R statistical software^39^. As we have reported previously^37^, MZ twins had slightly higher education than DZ twins, and therefore we allowed different means for MZ and DZ twins in the models.

We started the analyses by estimating the genetic and environmental variances in educational attainment in the whole dataset as well as in 10-year birth cohorts by first pooling data from all cohorts together and then stratifying them by the cultural–geographic region. In these analyses, we adjusted education by twin cohort because otherwise different educational systems between the countries and different education classifications between the surveys might have affected the results. Further, we adjusted for the effect of birth year in order to account for the effect of increasing educational level during the twentieth century in these cohorts^37^. The adjustments were done by calculating regression residuals of education by using birth year and the dummy variables of twin cohorts as independent variables in the regression models. We then analyzed temporal trends by pooling the birth cohorts into two broad time spans, 1900–1949 and 1950–1989, to increase power to detect differences in the genetic and environmental variation over the birth cohorts. We selected these cohorts because of the much higher education in the later born cohorts indicating extension in educational systems. We restricted these analyses to Europe and North America and Australia since older birth cohorts were not available in East Asia and in other countries. After these analyses by birth cohort, we conducted a second set of analyses and estimated genetic and environmental variance components separately in each twin cohort to confirm that the used classification did not hide any existing pattern. In these twin cohort specific analyses, we adjusted education for birth year separately in each twin cohort.

## Supplementary information

Supplementary Tables.

## Data Availability

This study is based on the re-analyses of original datasets from third parties. The data are available based on request if following the same rules and principles of CODATwins project followed also in this study. More information is available from the corresponding author.
