# Supplementary material for: Genetic and environmental variation in educational attainment: an individual-based analysis of 28 twin cohorts
Source: Sci Rep. 2020 Jul 29;10:12681. doi: 10.1038/s41598-020-69526-6 (PMC7391756; doi:10.1038/s41598-020-69526-6)
Supplement: Supplementary file 1 — Supplementary Tables. [file 41598_2020_69526_MOESM1_ESM.docx]

Genetic and environmental variation in educational attainment:

an individual-based analysis of 28 twin cohorts

Karri Silventoinen, Aline Jelenkovic, Reijo Sund, Antti Latvala, Chika Honda, Fujio Inui, Rie Tomizawa, Mikio Watanabe, Norio Sakai, Esther Rebato, Andreas Busjahn, Jessica Tyler, John L Hopper, Juan R Ordoñana, Juan F Sánchez-Romera, Lucia Colodro-Conde, Lucas Calais-Ferreira, Vinicius C Oliveira, Paulo H Ferreira, Emanuela Medda, Lorenza Nisticò, Virgilia Toccaceli, Catherine A Derom, Robert F Vlietinck, Ruth JF Loos, Sisira H Siribaddana, Matthew Hotopf, Athula Sumathipala, Fruhling Rijsdijk, Glen E Duncan, Dedra Buchwald, Per Tynelius, Finn Rasmussen, Qihua Tan, Dongfeng Zhang, Zengchang Pang, Patrik KE Magnusson, Nancy L Pedersen, Anna K Dahl Aslan, Amie E Hwang, Thomas M Mack, Robert F Krueger, Matt McGue, Shandell Pahlen, Ingunn Brandt, Thomas S Nilsen, Jennifer R Harris, Nicholas G Martin, Sarah E Medland, Grant W Montgomery, Gonneke Willemsen, Meike Bartels, Catharina EM van Beijsterveldt, Carol E Franz, William S Kremen, Michael J Lyons, Judy L Silberg, Hermine H Maes, Christian Kandler, Tracy L Nelson, Keith E Whitfield, Robin P Corley, Brooke M Huibregtse, Margaret Gatz, David A Butler, Adam D Tarnoki, David L Tarnoki, Hang A Park, Jooyeon Lee, Soo Ji Lee, Joohon Sung, Yoshie Yokoyama, Thorkild IA Sørensen, Dorret I Boomsma, Jaakko Kaprio

Supplementary table 1. Descriptive statistics by twin cohort.

| **Cohort name** | **Country** | **N of twins** | **% of females** | **Birth cohorts** | **Education** | | **N of educational categories** |
| --- | --- | --- | --- | --- | --- | --- | --- |
|  |  |  |  |  | **Mean** | **SD** |  |
| **Europe** |  |  |  |  |  |  |  |
| Berlin Twin Register | German | 160 | 52 | 1926–1987 | 12.4 | 3.27 | 5 |
| Bielefeld Longitudinal Study | German | 1065 | 78 | 1914–1969 | 12.3 | 4.41 | 7 |
| Netherlands Twin Cohort | Netherlands | 4694 | 67 | 1921–1989 | 14.1 | 2.54 | 5 |
| Finnish Older Twin Cohort | Finland | 28896 | 52 | 1880–1957 | 7.7 | 3.11 | 8 |
| FinnTwin12 | Finland | 1482 | 57 | 1983–1986 | 13.2 | 2.76 | 6 |
| FinnTwin16 | Finland | 3949 | 56 | 1974–1979 | 14.4 | 2.98 | 5 |
| East Flanders Prospective | Belgium | 459 | 51 | 1964–1975 | 13.6 | 2.36 | 7 |
| Hungarian Twin Registry | Hungarian | 262 | 70 | 1927–1985 | 14.2 | 3.94 | 6 |
| Italian Twin Registry | Italy | 5298 | 61 | 1917–1987 | 13.2 | 4.02 | 5 |
| Murcia Twin Registry | Spain | 2218 | 57 | 1940–1966 | 8.0 | 3.46 | 9 |
| Norwegian Twin Registry | Norway | 10690 | 53 | 1915–1960 | 11.6 | 3.00 | 4 |
| Swedish Young Male Twins | Sweden | 1184 | 0 | 1973–1978 | 14.0 | 2.03 | 7 |
| Swedish Twin Cohorts | Sweden | 39441 | 52 | 1900–1958 | 11.8 | 4.39 | 4 |
| **North America and Australia** |  |  |  |  |  |  |  |
| Twins Research Australian | Australia | 2328 | 77 | 1916–1989 | 15.0 | 2.58 | 13 |
| California Twin Program | USA | 24125 | 57 | 1908–1977 | 14.8 | 2.39 | 6 |
| Carolina African American | USA | 522 | 59 | 1910–1976 | 13.3 | 2.99 | >10 (exact years) |
| Colorado Twin Registry | USA | 1413 | 56 | 1979–1989 | 14.6 | 2.22 | 8 |
| Mid Atlantic Twin Registry | USA | 10425 | 65 | 1894–1987 | 14.2 | 3.06 | 6 |
| Minnesota Twin Registry | USA | 8395 | 55 | 1923–1958 | 14.0 | 2.37 | >10 (exact years) |
| Queensland Twin Register | Australia | 19030 | 61 | 1900–1989 | 14.2 | 2.21 | 7 |
| Vietnam Era Twin Registry | USA | 1237 | 0 | 1950–1950 | 13.9 | 2.10 | >10 (exact years) |
| NAS-NRC twin cohort | USA | 9173 | 0 | 1917–1927 | 13.5 | 3.10 | >10 (exact years) |
| Washington State Twin Registry | USA | 11987 | 62 | 1914–1988 | 14.8 | 2.36 | 8 |
| **East Asia** |  |  |  |  |  |  |  |
| Korean Twin-Family Register | South Korea | 1335 | 62 | 1934–1985 | 14.3 | 3.91 | 9 |
| Osaka University Aged Twin | Japan | 407 | 68 | 1924–1989 | 13.7 | 2.95 | >10 (exact years) |
| Qingdao Twin Registry | China | 996 | 52 | 1925–1982 | 10.4 | 3.50 | 6 |
| **Other regions** |  |  |  |  |  |  |  |
| Brazilian Twin Registry | Brazil | 133 | 73 | 1933–1992 | 16.4 | 2.58 | 7 |
| Sri Lanka Twin Registry | Sri Lanka | 2279 | 56 | 1925–1990 | 10.9 | 3.31 | 6 |

Supplementary table 2. The proportions of educational variation explained by additive genetic, shared environmental and unique environmental variances with 95% confidence intervals by birth cohort and gender.^1^

|  | **Additive genetic factors** | | | **Shared environment** | | | **Unique environment** | | |
| --- | --- | --- | --- | --- | --- | --- | --- | --- | --- |
|  | **a^2^** | **95% CI** | | **c^2^** | **95% CI** | | **e^2^** | **95% CI** | |
|  |  | **LL** | **UL** |  | **LL** | **LL** |  | **LL** | **UL** |
| **Men and women** |  |  |  |  |  |  |  |  |  |
| All | 0.42 | 0.40 | 0.44 | 0.32 | 0.30 | 0.33 | 0.26 | 0.26 | 0.27 |
| 1900–1909 | 0.12 | 0.00 | 0.25 | 0.62 | 0.51 | 0.72 | 0.26 | 0.22 | 0.32 |
| 1910–1919 | 0.37 | 0.30 | 0.44 | 0.43 | 0.36 | 0.49 | 0.21 | 0.19 | 0.23 |
| 1920–1929 | 0.43 | 0.39 | 0.48 | 0.33 | 0.29 | 0.37 | 0.24 | 0.23 | 0.25 |
| 1930–1939 | 0.41 | 0.36 | 0.46 | 0.32 | 0.27 | 0.36 | 0.27 | 0.26 | 0.29 |
| 1940–1949 | 0.47 | 0.43 | 0.51 | 0.26 | 0.23 | 0.29 | 0.27 | 0.26 | 0.28 |
| 1950–1959 | 0.38 | 0.34 | 0.41 | 0.36 | 0.33 | 0.39 | 0.26 | 0.25 | 0.27 |
| 1960–1969 | 0.27 | 0.22 | 0.33 | 0.39 | 0.33 | 0.44 | 0.34 | 0.33 | 0.36 |
| 1970–1979 | 0.37 | 0.30 | 0.44 | 0.32 | 0.25 | 0.38 | 0.31 | 0.29 | 0.33 |
| 1980–1989 | 0.62 | 0.44 | 0.76 | 0.11 | 0.00 | 0.28 | 0.27 | 0.23 | 0.31 |
| **Men** |  |  |  |  |  |  |  |  |  |
| All | 0.46 | 0.43 | 0.49 | 0.27 | 0.25 | 0.30 | 0.27 | 0.26 | 0.28 |
| 1900–1909 | 0.33 | 0.15 | 0.54 | 0.48 | 0.27 | 0.65 | 0.19 | 0.14 | 0.25 |
| 1910–1919 | 0.47 | 0.37 | 0.58 | 0.32 | 0.22 | 0.42 | 0.21 | 0.18 | 0.23 |
| 1920–1929 | 0.45 | 0.40 | 0.51 | 0.30 | 0.25 | 0.35 | 0.24 | 0.23 | 0.26 |
| 1930–1939 | 0.41 | 0.34 | 0.48 | 0.31 | 0.25 | 0.38 | 0.28 | 0.26 | 0.30 |
| 1940–1949 | 0.55 | 0.49 | 0.60 | 0.18 | 0.13 | 0.23 | 0.27 | 0.25 | 0.28 |
| 1950–1959 | 0.37 | 0.31 | 0.42 | 0.34 | 0.29 | 0.38 | 0.30 | 0.28 | 0.31 |
| 1960–1969 | 0.32 | 0.22 | 0.42 | 0.36 | 0.26 | 0.44 | 0.33 | 0.30 | 0.35 |
| 1970–1979 | 0.35 | 0.23 | 0.48 | 0.31 | 0.19 | 0.42 | 0.34 | 0.31 | 0.37 |
| 1980–1989 | 0.31 | 0.02 | 0.71 | 0.38 | 0.00 | 0.65 | 0.31 | 0.24 | 0.41 |
| **Women** |  |  |  |  |  |  |  |  |  |
| All | 0.38 | 0.36 | 0.40 | 0.36 | 0.34 | 0.38 | 0.26 | 0.25 | 0.26 |
| 1900–1909 | 0.05 | 0.00 | 0.17 | 0.69 | 0.58 | 0.76 | 0.26 | 0.22 | 0.32 |
| 1910–1919 | 0.25 | 0.16 | 0.33 | 0.54 | 0.46 | 0.62 | 0.21 | 0.19 | 0.24 |
| 1920–1929 | 0.39 | 0.32 | 0.46 | 0.39 | 0.32 | 0.45 | 0.22 | 0.20 | 0.24 |
| 1930–1939 | 0.41 | 0.34 | 0.48 | 0.32 | 0.26 | 0.38 | 0.27 | 0.25 | 0.29 |
| 1940–1949 | 0.39 | 0.35 | 0.44 | 0.34 | 0.30 | 0.38 | 0.27 | 0.25 | 0.28 |
| 1950–1959 | 0.38 | 0.34 | 0.42 | 0.38 | 0.34 | 0.42 | 0.24 | 0.23 | 0.25 |
| 1960–1969 | 0.25 | 0.18 | 0.32 | 0.40 | 0.33 | 0.47 | 0.35 | 0.33 | 0.37 |
| 1970–1979 | 0.38 | 0.30 | 0.47 | 0.32 | 0.24 | 0.40 | 0.30 | 0.28 | 0.32 |
| 1980–1989 | 0.67 | 0.51 | 0.77 | 0.08 | 0.00 | 0.23 | 0.21 | 0.25 | 0.31 |

^1^Restricted to participants 30 years of age or older

Supplementary table 3. The proportions of educational variation explained by additive genetic, shared environmental and unique environmental variances with 95% confidence intervals by birth cohort and cultural–geographic region in men.

|  | **Additive genetic factors** | | | **Shared environment** | | | **Unique environment** | | |
| --- | --- | --- | --- | --- | --- | --- | --- | --- | --- |
|  | **a^2^** | **95% CI** | | **c^2^** | **95% CI** | | **e^2^** | **95% CI** | |
|  |  | **LL** | **UL** |  | **LL** | **LL** |  | **LL** | **UL** |
| **Europe** |  |  |  |  |  |  |  |  |  |
| All | 0.43 | 0.40 | 0.47 | 0.29 | 0.26 | 0.32 | 0.28 | 0.27 | 0.29 |
| 1900–1909 | 0.43 | 0.24 | 0.67 | 0.47 | 0.23 | 0.65 | 0.10 | 0.06 | 0.16 |
| 1910–1919 | 0.47 | 0.00 | 0.63 | 0.26 | 0.12 | 0.71 | 0.28 | 0.22 | 0.35 |
| 1920–1929 | 0.32 | 0.21 | 0.43 | 0.41 | 0.31 | 0.50 | 0.28 | 0.24 | 0.32 |
| 1930–1939 | 0.34 | 0.25 | 0.43 | 0.35 | 0.27 | 0.42 | 0.31 | 0.28 | 0.34 |
| 1940–1949 | 0.50 | 0.44 | 0.57 | 0.22 | 0.15 | 0.28 | 0.28 | 0.26 | 0.30 |
| 1950–1959 | 0.37 | 0.30 | 0.43 | 0.35 | 0.29 | 0.41 | 0.28 | 0.26 | 0.30 |
| 1960–1969 | 0.59 | 0.46 | 0.68 | 0.11 | 0.03 | 0.21 | 0.30 | 0.26 | 0.36 |
| 1970–1979 | 0.54 | 0.40 | 0.68 | 0.17 | 0.04 | 0.29 | 0.29 | 0.26 | 0.33 |
| 1980–1989 | 0.56 | 0.26 | 0.80 | 0.18 | 0.00 | 0.46 | 0.25 | 0.19 | 0.34 |
| **North America and Australia** |  |  |  |  |  |  |  |  |  |
| All | 0.44 | 0.41 | 0.48 | 0.28 | 0.25 | 0.32 | 0.27 | 0.26 | 0.28 |
| 1900–1909 | 0.45 | 0.13 | 0.74 | 0.31 | 0.05 | 0.59 | 0.24 | 0.15 | 0.40 |
| 1910–1919 | 0.60 | 0.47 | 0.74 | 0.23 | 0.09 | 0.35 | 0.17 | 0.15 | 0.20 |
| 1920–1929 | 0.49 | 0.42 | 0.55 | 0.27 | 0.21 | 0.33 | 0.24 | 0.23 | 0.26 |
| 1930–1939 | 0.45 | 0.32 | 0.60 | 0.30 | 0.16 | 0.42 | 0.25 | 0.22 | 0.29 |
| 1940–1949 | 0.47 | 0.37 | 0.58 | 0.24 | 0.14 | 0.33 | 0.29 | 0.27 | 0.32 |
| 1950–1959 | 0.37 | 0.29 | 0.45 | 0.31 | 0.23 | 0.38 | 0.32 | 0.30 | 0.35 |
| 1960–1969 | 0.25 | 0.15 | 0.36 | 0.40 | 0.30 | 0.49 | 0.35 | 0.32 | 0.38 |
| 1970–1979 | 0.15 | 0.02 | 0.30 | 0.50 | 0.37 | 0.62 | 0.34 | 0.31 | 0.38 |
| 1980–1989 | 0.61 | 0.48 | 0.70 | 0.10 | 0.02 | 0.21 | 0.30 | 0.25 | 0.35 |
| **East Asia** |  |  |  |  |  |  |  |  |  |
| All | 0.22 | 0.05 | 0.47 | 0.48 | 0.24 | 0.65 | 0.30 | 0.25 | 0.35 |
| 1960–1969 | 0.44 | 0.10 | 0.82 | 0.38 | 0.01 | 0.70 | 0.19 | 0.13 | 0.26 |
| 1970–1979 | 0.27 | 0.05 | 0.59 | 0.34 | 0.03 | 0.57 | 0.39 | 0.32 | 0.47 |
| 1980–1989 | 0.61 | 0.01 | 0.79 | 0.00 | 0.00 | 0.62 | 0.39 | 0.21 | 0.68 |

Supplementary table 4. The proportions of educational variation explained by additive genetic, shared environmental and unique environmental variances with 95% confidence intervals by birth cohort and cultural–geographic region in women.

|  | **Additive genetic factors** | | | **Shared environment** | | | **Unique environment** | | |
| --- | --- | --- | --- | --- | --- | --- | --- | --- | --- |
|  | **a^2^** | **95% CI** | | **c^2^** | **95% CI** | | **e^2^** | **95% CI** | |
|  |  | **LL** | **UL** |  | **LL** | **LL** |  | **LL** | **UL** |
| **Europe** |  |  |  |  |  |  |  |  |  |
| All | 0.36 | 0.33 | 0.38 | 0.40 | 0.37 | 0.42 | 0.24 | 0.24 | 0.25 |
| 1900–1909 | 0.16 | 0.00 | 0.37 | 0.58 | 0.40 | 0.71 | 0.26 | 0.19 | 0.35 |
| 1910–1919 | 0.26 | 0.13 | 0.49 | 0.52 | 0.31 | 0.63 | 0.22 | 0.18 | 0.27 |
| 1920–1929 | 0.28 | 0.19 | 0.37 | 0.47 | 0.39 | 0.55 | 0.25 | 0.22 | 0.28 |
| 1930–1939 | 0.34 | 0.26 | 0.42 | 0.38 | 0.31 | 0.45 | 0.28 | 0.26 | 0.31 |
| 1940–1949 | 0.35 | 0.29 | 0.41 | 0.38 | 0.33 | 0.43 | 0.27 | 0.25 | 0.29 |
| 1950–1959 | 0.41 | 0.36 | 0.46 | 0.39 | 0.34 | 0.43 | 0.21 | 0.19 | 0.22 |
| 1960–1969 | 0.19 | 0.04 | 0.35 | 0.47 | 0.32 | 0.60 | 0.34 | 0.30 | 0.38 |
| 1970–1979 | 0.44 | 0.33 | 0.56 | 0.27 | 0.16 | 0.38 | 0.28 | 0.26 | 0.31 |
| 1980–1989 | 0.69 | 0.52 | 0.75 | 0.01 | 0.00 | 0.17 | 0.30 | 0.25 | 0.36 |
| **North America and Australia** |  |  |  |  |  |  |  |  |  |
| All | 0.32 | 0.29 | 0.35 | 0.37 | 0.34 | 0.40 | 0.31 | 0.30 | 0.32 |
| 1900–1909 | 0.00 | 0.00 | 0.14 | 0.74 | 0.60 | 0.80 | 0.26 | 0.20 | 0.34 |
| 1910–1919 | 0.19 | 0.07 | 0.33 | 0.60 | 0.47 | 0.71 | 0.21 | 0.18 | 0.25 |
| 1920–1929 | 0.45 | 0.34 | 0.58 | 0.32 | 0.20 | 0.43 | 0.22 | 0.20 | 0.25 |
| 1930–1939 | 0.41 | 0.28 | 0.56 | 0.28 | 0.14 | 0.40 | 0.31 | 0.27 | 0.34 |
| 1940–1949 | 0.37 | 0.29 | 0.46 | 0.33 | 0.25 | 0.40 | 0.30 | 0.28 | 0.33 |
| 1950–1959 | 0.30 | 0.23 | 0.37 | 0.37 | 0.30 | 0.43 | 0.33 | 0.31 | 0.35 |
| 1960–1969 | 0.31 | 0.23 | 0.40 | 0.32 | 0.25 | 0.39 | 0.36 | 0.34 | 0.39 |
| 1970–1979 | 0.24 | 0.14 | 0.34 | 0.40 | 0.31 | 0.49 | 0.36 | 0.33 | 0.39 |
| 1980–1989 | 0.43 | 0.29 | 0.59 | 0.27 | 0.12 | 0.41 | 0.29 | 0.26 | 0.33 |
| **East Asia** |  |  |  |  |  |  |  |  |  |
| All | 0.37 | 0.20 | 0.58 | 0.42 | 0.21 | 0.59 | 0.21 | 0.18 | 0.24 |
| 1960-1969 | 0.11 | 0.00 | 0.49 | 0.63 | 0.26 | 0.78 | 0.26 | 0.20 | 0.34 |
| 1970-1979 | 0.44 | 0.20 | 0.75 | 0.34 | 0.04 | 0.58 | 0.22 | 0.18 | 0.27 |
| 1980-1989 | 0.80 | 0.50 | 0.88 | 0.00 | 0.00 | 0.27 | 0.20 | 0.12 | 0.35 |

Supplementary table 5. The proportions of educational variation explained by additive genetic, shared environmental and unique environmental variances with 95% confidence intervals by birth cohort in men and women.

|  | **Additive genetic factors** | | **Shared environment** | | **Unique environment** | |
| --- | --- | --- | --- | --- | --- | --- |
|  | **a^2^** | **95% CI** | **c^2^** | **95% CI** | **e^2^** | **95% CI** |
| **Europe** |  |  |  |  |  |  |
| Berlin Twin Register | 0.00 | 0.00–0.64 | 0.68 | 0.24–0.79 | 0.32 | 0.21–0.46 |
| Bielefeld Longitudinal | 0.38 | 0.14–0.67 | 0.31 | 0.02–0.53 | 0.32 | 0.27–0.37 |
| Netherlands Twin Cohort | 0.56 | 0.43–0.70 | 0.13 | 0.00–0.26 | 0.31 | 0.28–0.33 |
| Finnish Older Twin Cohort | 0.45 | 0.42–0.48 | 0.36 | 0.33–0.39 | 0.19 | 0.18–0.20 |
| FinnTwin12 | 0.55 | 0.26–0.73 | 0.12 | 0.00–0.37 | 0.33 | 0.26–0.42 |
| FinnTwin16 | 0.43 | 0.29–0.57 | 0.27 | 0.14–0.40 | 0.30 | 0.27–0.34 |
| East Flanders Prospective | 0.72 | 0.46–0.79 | 0.00 | 0.00–0.24 | 0.28 | 0.21–0.37 |
| Hungarian Twin Registry | 0.57 | 0.17–0.85 | 0.22 | 0.00–0.60 | 0.21 | 0.15–0.30 |
| Italian Twin Registry | 0.32 | 0.23–0.41 | 0.43 | 0.34–0.52 | 0.25 | 0.23–0.27 |
| Murcia Twin Registry | 0.54 | 0.39–0.71 | 0.25 | 0.09–0.38 | 0.21 | 0.18–0.25 |
| Norwegian Twin Registry | 0.56 | 0.49–0.63 | 0.22 | 0.16–0.28 | 0.22 | 0.20–0.24 |
| Swedish Young Male Twins | 0.62 | 0.38–0.70 | 0.03 | 0.00–0.24 | 0.35 | 0.30–0.42 |
| Swedish Twin Cohorts | 0.40 | 0.35–0.45 | 0.25 | 0.20–0.29 | 0.35 | 0.33–0.37 |
| **North America and Australia** |  |  |  |  |  |  |
| Australian Twin Registry | 0.49 | 0.30–0.62 | 0.09 | 0.00–0.27 | 0.42 | 0.38–0.47 |
| California Twin Program | 0.33 | 0.29–0.38 | 0.35 | 0.31–0.39 | 0.32 | 0.30–0.33 |
| Carolina African American | 0.31 | 0.07–0.57 | 0.43 | 0.20–0.62 | 0.26 | 0.19–0.35 |
| Colorado Twin Registry | 0.73 | 0.50–0.77 | 0.00 | 0.00–0.21 | 0.27 | 0.23–0.33 |
| Mid Atlantic Twin Registry | 0.44 | 0.38–0.51 | 0.36 | 0.30–0.42 | 0.20 | 0.18–0.21 |
| Minnesota Twin Registry | 0.44 | 0.35–0.53 | 0.30 | 0.22–0.38 | 0.26 | 0.24–0.29 |
| Queensland Twin Register | 0.33 | 0.26–0.39 | 0.29 | 0.24–0.35 | 0.38 | 0.36–0.40 |
| Vietnam Era Twin Registry | 0.54 | 0.33–0.69 | 0.10 | 0.00–0.28 | 0.36 | 0.31-0.43 |
| NAS-NRC twin cohort | 0.48 | 0.41–0.54 | 0.28 | 0.21–0.34 | 0.25 | 0.23–0.26 |
| Washington State Twin Registry | 0.37 | 0.29–0.45 | 0.28 | 0.21–0.36 | 0.35 | 0.33–0.37 |
| **East Asia** |  |  |  |  |  |  |
| Korean Twin-Family Register | 0.32 | 0.11–0.58 | 0.38 | 0.12–0.58 | 0.30 | 0.26–0.34 |
| Osaka University Aged Twin | 0.62 | 0.30–0.71 | 0.00 | 0.00–0.29 | 0.38 | 0.29–0.49 |
| Qingdao Twin Registry | 0.38 | 0.20–0.61 | 0.46 | 0.23–0.63 | 0.16 | 0.13–0.19 |
| **Other regions** |  |  |  |  |  |  |
| Sri Lanka Twin Registry | 0.40 | 0.24–0.59 | 0.34 | 0.17–0.49 | 0.25 | 0.22–0.30 |

Supplementary table 6. The proportions of educational variation explained by additive genetic, shared environmental and unique environmental variances with 95% confidence intervals by birth cohort in men.

|  | **Additive genetic factors** | | **Shared environment** | | **Unique environment** | |
| --- | --- | --- | --- | --- | --- | --- |
|  | **a^2^** | **95% CI** | **c^2^** | **95% CI** | **e^2^** | **95% CI** |
| **Europe** |  |  |  |  |  |  |
| Berlin Twin Register | 0.00 | 0.00–0.64 | 0.69 | 0.04–0.82 | 0.31 | 0.18–0.51 |
| Bielefeld Longitudinal | 0.41 | 0.00–0.59 | 0.02 | 0.00–0.43 | 0.57 | 0.40–0.78 |
| Netherlands Twin Cohort | 0.64 | 0.50–0.71 | 0.04 | 0.00–0.16 | 0.32 | 0.27–0.37 |
| Finnish Older Twin Cohort | 0.47 | 0.42–0.52 | 0.32 | 0.27–0.36 | 0.21 | 0.20–0.23 |
| FinnTwin12 | 0.36 | 0.02–0.76 | 0.36 | 0.00–0.64 | 0.29 | 0.21–0.40 |
| FinnTwin16 | 0.56 | 0.35–0.77 | 0.17 | 0.00–0.36 | 0.26 | 0.22–0.33 |
| East Flanders Prospective | 0.71 | 0.19–0.84 | 0.05 | 0.00–0.53 | 0.24 | 0.16–0.36 |
| Hungarian Twin Registry | 0.80 | 0.17–0.93 | 0.07 | 0.00–0.68 | 0.13 | 0.07–0.26 |
| Italian Twin Registry | 0.30 | 0.15–0.47 | 0.42 | 0.26–0.56 | 0.28 | 0.25–0.32 |
| Murcia Twin Registry | 0.57 | 0.33–0.81 | 0.21 | 0.00–0.42 | 0.22 | 0.17–0.29 |
| Norwegian Twin Registry | 0.64 | 0.54–0.75 | 0.14 | 0.03–0.24 | 0.22 | 0.19–0.24 |
| Swedish Young Male Twins | 0.62 | 0.38–0.70 | 0.03 | 0.00–0.24 | 0.35 | 0.30–0.42 |
| Swedish Twin Cohorts | 0.43 | 0.35–0.50 | 0.22 | 0.15–0.28 | 0.35 | 0.33–0.38 |
| **North America and Australia** |  |  |  |  |  |  |
| Australian Twin Registry | 0.57 | 0.24–0.66 | 0.00 | 0.00–0.31 | 0.43 | 0.34–0.54 |
| California Twin Program | 0.37 | 0.29–0.44 | 0.33 | 0.26–0.39 | 0.30 | 0.28–0.33 |
| Carolina African American | 0.02 | 0.00–0.35 | 0.66 | 0.33–0.77 | 0.32 | 0.21–0.47 |
| Colorado Twin Registry | 0.51 | 0.25–0.62 | 0.01 | 0.00–0.23 | 0.48 | 0.38–0.61 |
| Mid Atlantic Twin Registry | 0.58 | 0.47–0.71 | 0.24 | 0.12–0.35 | 0.18 | 0.16–0.20 |
| Minnesota Twin Registry | 0.40 | 0.26–0.55 | 0.30 | 0.15–0.43 | 0.30 | 0.27–0.34 |
| Queensland Twin Register | 0.32 | 0.22–0.44 | 0.30 | 0.20–0.39 | 0.38 | 0.35–0.41 |
| Vietnam Era Twin Registry | 0.54 | 3.30–0.69 | 0.10 | 0.00–0.28 | 0.36 | 3.07–0.43 |
| NAS-NRC twin cohort | 0.48 | 0.41–0.54 | 0.28 | 0.21–0.34 | 0.25 | 0.23–0.26 |
| Washington State Twin Registry | 0.45 | 0.33–0.59 | 0.24 | 0.11–0.35 | 0.31 | 0.28–0.34 |
| **East Asia** |  |  |  |  |  |  |
| Korean Twin-Family Register | 0.16 | 0.00–0.60 | 0.47 | 0.05–0.68 | 0.37 | 0.30–0.46 |
| Osaka University Aged Twin | 0.56 | 0.11–0.75 | 0.02 | 0.00–0.38 | 0.42 | 0.25–0.71 |
| Qingdao Twin Registry | 0.14 | 0.02–0.39 | 0.69 | 0.44–0.81 | 0.18 | 0.14–0.23 |
| **Other regions** |  |  |  |  |  |  |
| Sri Lanka Twin Registry | 0.43 | 0.15–0.74 | 0.28 | 0.00–0.52 | 0.30 | 0.23–0.38 |

Supplementary table 7. The proportions of educational variation explained by additive genetic, shared environmental and unique environmental variances with 95% confidence intervals by birth cohort in women.

|  | **Additive genetic factors** | | **Shared environment** | | **Unique environment** | |
| --- | --- | --- | --- | --- | --- | --- |
|  | **a^2^** | **95% CI** | **c^2^** | **95% CI** | **e^2^** | **95% CI** |
| **Europe** |  |  |  |  |  |  |
| Berlin Twin Register | 0.00 | 0.00–0.67 | 0.67 | 0.00–0.81 | 0.33 | 0.19–0.55 |
| Bielefeld Longitudinal Study | 0.31 | 0.10–0.57 | 0.44 | 0.18–0.63 | 0.25 | 0.21–0.31 |
| Netherlands Twin Cohort | 0.48 | 0.34–0.63 | 0.22 | 0.07–0.35 | 0.31 | 0.28–0.34 |
| Finnish Older Twin Cohort | 0.42 | 0.38–0.46 | 0.41 | 0.37–0.44 | 0.17 | 0.16–0.18 |
| FinnTwin12 | 0.59 | 0.25–0.72 | 0.05 | 0.00–0.34 | 0.36 | 0.28–0.48 |
| FinnTwin16 | 0.31 | 0.13–0.50 | 0.36 | 0.18–0.51 | 0.33 | 0.28–0.38 |
| East Flanders Prospective | 0.51 | 0.00–0.79 | 0.18 | 0.00–0.65 | 0.31 | 0.21–0.45 |
| Hungarian Twin Registry | 0.48 | 0.04–0.83 | 0.28 | 0.00–0.69 | 0.24 | 0.16–0.36 |
| Italian Twin Registry | 0.33 | 0.22–0.45 | 0.44 | 0.33–0.54 | 0.23 | 0.21–0.26 |
| Murcia Twin Registry | 0.50 | 0.32–0.72 | 0.28 | 0.08–0.45 | 0.21 | 0.17–0.26 |
| Norwegian Twin Registry | 0.44 | 0.36–0.52 | 0.34 | 0.26–0.41 | 0.22 | 0.20–0.24 |
| Swedish Twin Cohorts | 0.38 | 0.31–0.45 | 0.28 | 0.22–0.33 | 0.35 | 0.33–0.37 |
| **North America and Australia** |  |  |  |  |  |  |
| Australian Twin Registry | 0.46 | 0.23–0.63 | 0.12 | 0.00–0.34 | 0.42 | 0.37–0.47 |
| California Twin Program | 0.31 | 0.25–0.37 | 0.36 | 0.31–0.42 | 0.33 | 0.31–0.34 |
| Carolina African American | 0.50 | 0.21–0.80 | 0.29 | 0.02–0.55 | 0.20 | 0.14–0.30 |
| Colorado Twin Registry | 0.36 | 0.14–0.65 | 0.38 | 0.10–0.58 | 0.26 | 0.21–0.33 |
| Mid Atlantic Twin Registry | 0.36 | 0.30–0.44 | 0.43 | 0.36–0.49 | 0.21 | 0.19–0.23 |
| Minnesota Twin Registry | 0.49 | 0.38–0.61 | 0.29 | 0.18–0.39 | 0.22 | 0.20–0.25 |
| Queensland Twin Register | 0.33 | 0.25–0.41 | 0.29 | 0.22–0.36 | 0.38 | 0.36–0.40 |
| Washington State Twin Registry | 0.29 | 0.20–0.40 | 0.33 | 0.24–0.42 | 0.37 | 0.35–0.40 |
| **East Asia** |  |  |  |  |  |  |
| Korean Twin-Family Register | 0.41 | 0.15–0.76 | 0.33 | 0.00–0.58 | 0.26 | 0.22–0.31 |
| Osaka University Aged Twin | 0.00 | 0.00–0.29 | 0.65 | 0.35–0.74 | 0.35 | 0.26–0.46 |
| Qingdao Twin Registry | 0.52 | 0.28–0.75 | 0.34 | 0.11–0.57 | 0.15 | 0.11–0.19 |
| **Other regions** |  |  |  |  |  |  |
| Brazilian Twin Registry | 0.34 | 0.00–0.82 | 0.36 | 0.00–0.82 | 0.30 | 0.17–0.50 |
| Sri Lanka Twin Registry | 0.38 | 0.19–0.61 | 0.39 | 0.17–0.57 | 0.23 | 0.19–0.28 |
